# Marking *Drosophila suzukii* (Diptera: Drosophilidae) with Fluorescent Dusts

**DOI:** 10.3390/insects11030152

**Published:** 2020-03-01

**Authors:** Rik Clymans, Vincent Van Kerckvoorde, Tim Beliën, Dany Bylemans, Patrick De Clercq

**Affiliations:** 1 Zoology Department, Research Centre for Fruit Cultivation (pcfruit npo), Fruittuinweg 1, B-3800 Sint-Truiden, Belgium; Rik.clymans@pcfruit.be (R.C.); vincent.vankerckvoorde@pcfruit.be (V.V.K.); tim.belien@pcfruit.be (T.B.); dany.bylemans@pcfruit.be (D.B.); 2 Department of Biosystems, KU Leuven, Decroylaan 42, B-3001 Heverlee, Belgium; 3 Department of Plants and Crops, Faculty of Bioscience Engineering, Ghent University, Coupure Links 653, B-9000 Ghent, Belgium

**Keywords:** *Drosophila suzukii*, insect marking, mark-release-recapture, mark-recapture, dispersal, trapping, RadGlo^®^, DayGlo^®^, modelling, behaviour

## Abstract

The marking of *Drosophila suzukii* can be an important instrument for studying the ecology and behaviour of this economically important fruit pest, aiding the development of new Integrated Pest Management (IPM) tools or strategies. There is, however, a need for a cost-effective methodology that provides an easily detectable and stable mark. Whereas fluorescent pigment powders are often used in entomological research, the pigments (series, dyes), application techniques, or doses need to be evaluated for each studied species in terms of their efficacy and possible adverse effects on the performance of the insect. The effectiveness of different application techniques and dyes (RadGlo^®^ TP-series) and their effect on the survival of adult *D. suzukii* were investigated in the laboratory. Furthermore, the influence of the marking on the behaviour of the flies was examined in laboratory trap assays (olfaction) and a field recapture study (general orientation). The persistence and detectability of the marks was evaluated both on living flies (for different application techniques) and dead flies under trapping/storage conditions. The use of fluorescent powders to mark *D. suzukii* flies yielded a clearly detectable and highly persistent mark, without any adverse effects on the survival and behaviour of the flies.

## 1. Introduction

The marking of insects has been of interest for more than 100 years [1,2,3] in insect ecology and applied entomology. Mark-release-recapture (MRR), mark-recapture (MR) and mark-capture (MC) studies are used to investigate insect dispersal and/or population dynamics and to assess or model the interaction with trapping devices. Marking is also used to facilitate the differentiation of individuals or treatment groups in laboratory, semi-field, or field experiments. Moreover, sterile insect technique (SIT) programmes include a marking step in order to evaluate the density and dispersal of released insects. The indirect marking of insects via possible hosts or resources (MC studies) can support studies on trophic interactions, and the use of or preference for resources. In general, the marker should be inexpensive, easily applied, persistent, and unequivocally and easily detected. Moreover, it should not interfere with the development, longevity, or behaviour of the insect. There are many different methods for marking insects and it is highly dependent on the species and type of experiment/application which of these is fit for purpose. An overview of methods is given by Hagler and Jackson [4].

The spotted wing Drosophila, *Drosophila suzukii* Matsumura (Diptera: Drosophilidae), native to Asia [5,6,7], has become a worldwide invasive pest of soft-skinned fruit crops over the last decade [8,9,10,11,12,13,14]. Studies on the ecology and behaviour of the species can yield new insights allowing the improvement or development of management strategies. Various methods for marking *D. suzukii* flies have been proposed and further optimised. An immunomarking technique (i.e., the use of proteins as markers and detection by enzyme-linked immuno-sorbent assays (ELISA) [15]) for both topical application and acquisition of residues on leaves was tested and optimised by Klick et al. in a series of laboratory and semi-field experiments [16]. What makes this technique unique is that vast areas can be sprayed with inexpensive proteins and that detection by ELISA is fairly sensitive to the acquisition of leaf residues. The disadvantages of immunomarking are the possibility of both false positives and false negatives [17], the need for individual analyses of specimens, and the costs and labour required for detection. Trace elements and stable isotopes can be administered to plants to act as systemic markers for herbivorous insects, an approach that was evaluated for *D. suzukii* with Rb and ^15^N. [18]. The added value of these systemic markers lies in the fact that larvae are self-marked while developing inside the host fruits, enabling the linkage of captured adults to their source of larval development [18]. Methods of self-marking are particularly interesting for MC studies in which whole areas or plants are marked, instead of marking the (captured/reared) insects before release. For MR and MRR studies, it is possible and more ideal to mark groups of flies with a visual marker to facilitate detection.

Daylight fluorescent pigments (fluorescent dusts) have been shown to be suitable markers for other Diptera: they have been thoroughly evaluated for dusting mosquito adults [19,20,21] and are used in the self-marking of Tephritidae on emergence, by locking pigment in the ptilinal suture after ptilinum collapse [22,23,24,25]. For *D. suzukii* or any other *Drosophila* species, however, the use of modern-day fluorescent dusts (i.e., organic dyes in a resin matrix, ground to fine particles [26,27,28]) was never thoroughly evaluated. Rice et al. [29], Kirkpatrick et al. [30], and Drummond et al. [31] did make use of these fluorescent pigments for marking *D. suzukii* but did either not justify their methodology or simply referred to method evaluations for other Diptera [19,24,32]. Drummond et al. [31], on the other hand, did perform a single unreplicated field evaluation of the effects of marking on recapture and dispersal. Studies on other *Drosophila* spp. usually failed to refer to method evaluations and only assumed that there were no significant adverse effects [33,34,35,36,37,38,39] or cited evaluations of unspecified [40] or outdated pigments (inorganic, CdS and ZnS based, “Helecon” pigments, United States Radium Corporation, U.S.) [41,42,43]. Therefore, there is a need for a more thorough assessment of the effect of modern-day, specified, fluorescent pigments for the marking of *D. suzukii* on the survival and behaviour of the flies. Moreover, it is warranted to evaluate the persistence of the marker and optimise the method of application. Like in studies with mosquitoes, the pigments are commonly applied on adult *Drosophila* flies by shaking the flies in a small amount of powder [31,43,44,45,46], through self-marking by enclosing the flies in dusted vials [34,47], by dusting them with a bulb duster/powder insufflator [29,30,32,48], or through unspecified means [33,35,36,37,38,39,40,41,42,49,50,51]. In Drosophilidae, it is also possible to use the aforementioned self-marking methodology for Tephritidae as a ptilinum is present [52,53]. This technique of marking upon emergence is, however, labour intensive in drosophilid flies given that the isolation of pupae is required and therefore this method is probably most useful when minimising the amount of pigments is desirable for economic reasons, like in SIT programmes [53].

Modern-day, daylight fluorescent pigments with an ultraviolet (UV) response typically consist of a dyed (commonly with rhodamines, aminonaphthalimides, and coumarins [26]) and mechanically-ground (toluene)sulfonamide–melamine–(para)formaldehyde resin matrix and are used in the manufacturing of inks and plastics. These pigments are resistant to most solvents and have a relatively good lightfastness (UV stabilised). There is a trend towards the use of formaldehyde-free pigments, where alternative resins (polyurethane, polyamide or polyester) are used as a matrix [26,27,28]. Frequently used fluorescent pigment products in insect marking are DayGlo^®^ (Day-Glo Color Corp., Cleveland Ohio U.S.A.), RadGlo^®^ (Radiant Color NV, Houthalen-Helchteren, Belgium), and SWADA (Dane Color UK Ltd., Stalybridge, UK).

The aim of the present study was to evaluate, both under laboratory and field conditions, the suitability of fluorescent pigment dusts to simultaneously mark groups of adult *D. suzukii* flies.

## 2. Materials and Methods

### 2.1. Pigment Application Methods

**Figure 1:**
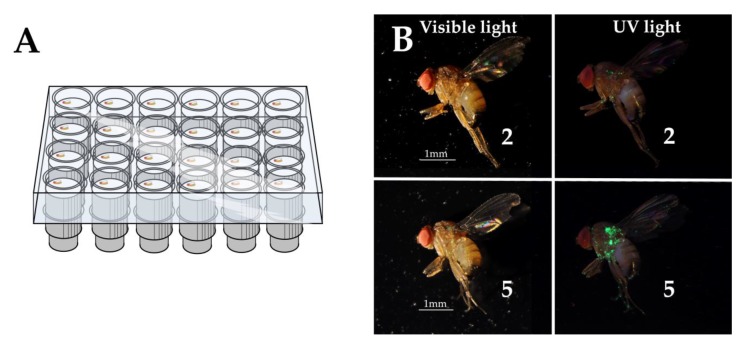
*D. suzukii* flies were individually kept in observation cells (**A**) to investigate the effect of marking on survival as well as to assess the marking efficiency and persistence for different marking techniques using Radglo^®^ TP-40: Chartreuse. An illustration of the scoring system (here scores 2 and 5) applied to calculate marking efficiency is given under both visible light and UV light (**B**).

The culture of *D. suzukii* originated from infested fruits collected in a blackberry field (50°46.243′ N, 5°9.665′ E, Sint-Truiden, Belgium) that were collected less than 12 months prior to the experiment. The insects were reared using a cornmeal-sugar-yeast diet [54] in tubes (50 mL, transparent PP, skirted centrifuge tube, nerbe plus GmbH) stoppered with foam stoppers (Ceaprenstop, Ø 36 mm, Greiner Bio-One™) and kept in an incubator at 25 ± 1 °C, 65 ± 5% relative humidity (RH) and a 12:12 h L:D photoperiod. In order to compare different methods for applying fluorescent pigment powders to adult *D. suzukii*, a single fluorescent pigment was applied using three different techniques as compared with a mock-treatment (no pigment, same handling). The pigment was Radglo^®^ TP-40 (Radiant Color NV, Houthalen-Helchteren, Belgium), which is a Chartreuse (yellow-green) coloured daylight and UV responsive fluorescent pigment (dyed, thermoset, sulphonamide-melamine-paraformaldehyde resin) with an average particle size of ca. 5.0 μm. The three application techniques tested were: dusting with a syringe, dusting with a bulb duster, and shaking in a vial containing a small amount of pigment. Seven-day-old flies were selected from the rearing and placed in groups of six (sex ratio 50:50) in a 50 mL centrifuge tube. The bottom of the tube was fitted with a cellulose acetate plug (Flugs™, MLS) saturated with 10% sugar solution and the tube was stoppered with a foam stopper. Flies were transferred to the test tubes with an aspirator (unanesthetised). Eight tubes, containing three males and three females, were randomly assigned to each treatment group (i.e., “Control”, “Syringe”, “Bulb duster”, and “Shaking”) and the flies in each tube were marked simultaneously, thus the four treatments were applied on groups of six flies (sex ratio 50:50) and this was repeated eight times. Prior to marking, flies were cold-anesthetised by placing them for about 30 s in a centrifuge tube on ice. The cold-anesthetised flies were then placed in a clean 30 mL glass vial (snap cap type, height 6 cm, outer Ø 3 cm, inner Ø of opening 2 cm) for marking. Dusting by syringe was done with a 5 mL syringe (3 parts syringe Romed^®^ Holland, Van Oostveen Medical B.V), loaded with 1 mg of pigment in the 21 G syringe needle (BD Plastipak™). The needle tip was held in the centre of the vial opening and 5 mL of air was administered at once, resulting in a deposition of 0.81 ± 0.14 mg (n = 10). Dusting by bulb duster was done by using a 65 mL atomiser bulb with pressure valve (Deutsch and Neumann) connected to a pigment reservoir (50 mL, transparent PP, skirted centrifuge tube, nerbe plus GmbH). The outlet tube (PVC, 4 mm inner Ø) was held in the centre of the vial opening and one puff was administered, resulting in a deposition of 9.85 ± 2.56 mg (n = 10). Dusting by shaking was done by placing a spatula tip of pigment (~20 mg) in the glass vial, then adding the cold-anesthetised flies and swirling the vial for about 10 s. The control was a mock treatment where flies underwent the same handling and anaesthesia procedure. The different dusting techniques are aligning with different coverage rates or “doses” of pigment powder. The shaking method resulted in near complete coverage of the insect. The bulb duster deposited considerably less, but still about ten times the amount of the syringe. After marking, the flies were immediately transferred back to their sugar solution substrate tubes. These tubes were placed in an incubator (25 ± 1 °C, 65 ± 5% RH, 12:12 h L:D) for 24 h to allow the marked flies to groom off excess pigment powder. Thereafter, the flies were cold-anesthetised again and were confined in individual cells for further observation. These cells were created by plugging the wells of 24-well plates (clear, flat bottom, sterile, TC-treated, VWR International) with upside-down push caps for 5 mL plastic vials (PE, opaque, hollow, 27 mm long with a 7 mm part to push in the vial and a 20 mm straight ribbed edged grip, the latter with an outer and inner Ø of 16 and 13 mm, respectively). The push caps fit tightly in the wells, but their ribbed edges allow ventilation. Each push cap was fitted with a cotton wool ball saturated with a 10% sugar solution. Inverting the well plates creates about 1 cm of space, allowing the flies to move freely while they can be easily observed on top of the cotton wool. A visual representation of the observation cells is given in Figure 1A. Flies of each treatment and sex were randomly assigned to individual wells of a well plate, resulting in eight 24-well plates, each with six individuals (3 males and 3 females) for the four treatment groups. The well plates were kept in an incubator (25 ± 1 °C, 65 ± 5% RH, 12:12 h L:D) for the duration of the experiment.

#### 2.1.1. Effect on Survival

In order to assess whether exposure to the fluorescent pigment had an effect on the survival of male or female flies and whether a dose response could be noted, 24 individuals per treatment group and sex were daily inspected until all individuals (sugar water fed) had died.

#### 2.1.2. Marking Efficiency and Persistence

The same individuals confined in the observation cells were assessed daily for marking efficiency and the persistence of the marks over a 15 day period. A marking efficiency index was determined per sex and per cluster of simultaneously treated flies on every observation day by scoring each individual and using the Townsend-Heuberger formula [55]: marking efficiency (%) = $\left(\sum^{\text{​}}(n*\;v\right)/\left(m*\;N\right))*100$ with *n* = the number of flies with each score value; *v* = the score value; *m* = the highest possible score (here: 5); and *N* = the total amount of scored flies. Assessments were done by placing the observation cells under a stereomicroscope with UV light (UV compact fluorescent lamp, 25 W, BeamZ). The given scores ranged from 0 to 5: 0 = no pigment; 1 = traces of pigment; 2 = ungroomed body surfaces homogeneously covered with individual particles—little surface covered; 3 = ungroomed surfaces homogeneously covered with individual particles—more surface covered; 4 = ungroomed surfaces covered with compacted powder (clumps)—little surface covered; 5 = ungroomed surfaces covered with compacted powder (clumps)—more surface covered. Figure 1B illustrates scores “2” and “5” both in visual and UV light and the whole set of scores is presented in Figure S1.

### 2.2. Effects of Colour on Survival

The possible effects of the dye used in the pigment (another colour means another organic dye was used to colour the resin) on survival was investigated by using the “Shaking” application technique on seven-day-old males and females. The additional tested colours were: Orange (TP-43), Magenta, (TP-48) and Blue (TP-49) from the same product series as the Radglo^®^ TP-40 (Chartreuse, already evaluated in Section 2.1.). These three colours were compared to a mock treatment using the same fly culture, protocol and number of replicates as described in Section 2.1.

### 2.3. Effects on Behaviour

#### 2.3.1. Olfaction of Marked Flies in Laboratory Trap Assays

**Figure 2:**
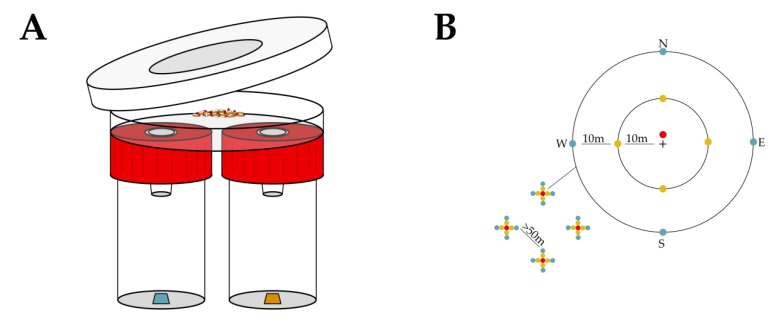
The effect of marking with fluorescent pigments on the behaviour of *D. suzukii* was evaluated both in the laboratory and field. In the laboratory, the effect on olfaction was investigated using trap assays: releasing both marked and unmarked flies in test units to allow a choice between water as a control and ACV (**A**). In the field, the effect on overall orientation was evaluated by placing traps (providing both visual and olfactory cues) at 1, 10 and 20 m from a central release point (represented by “+”), in four replicates (grids of traps depicted with indication of their spacing and cardinal direction) and assessing recapture of the releases marked and unmarked flies (**B**).

A first assessment of the possible effect of the “Shaking” marking technique on the behaviour of *D. suzukii* adult males and females was done in the laboratory using dual choice trap assays, hence focusing on the possible disturbance of olfaction. Flies of 4–6 days old were cold-anesthetised and placed per 20 (sex ratio 50:50) in a sugar solution substrate tube (as described in Section 2.1). The flies in half of the tubes were marked with Radglo^®^ TP-40 (Chartreuse) using the “Shaking” technique (as described in Section 2.1) and the other half received a mock treatment without pigment. After marking, flies were placed back in the tubes and transferred to an incubator (25 ± 1 °C, 65 ± 5% RH, 12:12 h L:D). The flies were allowed to groom off excess dust for 24 h, but 19 h after treatment all flies were transferred to new tubes containing only water in order to create a 5 h starvation period prior to the trap assays. For the experiment, 12 replicates of the trap assay unit depicted in Figure 2A were used. The units were constructed by punching two 1 cm Ø holes through the bottom of a Petri dish (90 mm Ø, clear, PS, Gosselin^™^), 3 cm apart, and punching equal size holes through the centre of two vial screw caps (PE, red, from 60 mL PP vials, Corning^®^ Gosselin^™^ S.A.S.). In the holes, 1.5 mL centrifuge tubes (PP, graduated, natural, Greiner Bio-One) were inserted. The centrifuge tubes, with their caps removed and tips cut to create a 5 mm hole, served as funnel entries. These funnel entries prevented the flies from turning back once a choice was made. On each trap assay unit two 60 mL vials (PP, transparent, 33 mm Ø, Corning^®^ Gosselin^™^ S.A.S.) were screwed containing a 1 cm^2^ piece of filter paper (grade 41, ashless, Whatman^®^) loaded 1 h prior to inserting the test flies with 10 µL of either apple cider vinegar (ACV) (cider vinegar 5% acidity, Burg, Vinaigrerie Fuchs, La Tremblade, France) or water. In each unit, 20 marked and 20 unmarked flies (sex ratio 50:50) were placed in the Petri dish at the furthest point of both holes using cold-anaesthesia, an aspirator, and a guiding tube. The Petri dish was then closed with a ventilated (4 cm Ø central 53 µm pore nylon mesh) lid (10 cm Ø, transparent, PS, SPL Life Sciences Co., Ltd.) secured to the dish bottom with two pieces of transparent tape. All units were placed in an incubator (25 ± 1 °C, 65 ± 5% RH, 12:12 h L:D). After 24 h the units were placed in a freezer to kill all flies in order to facilitate the assessment of choices made by the flies. For each combination of treatment and sex, a preference index (PI) was calculated for each trap assay unit with PI = (the number of flies in the ACV-loaded vial − the number of flies in the water-loaded vial)/(the number of flies in the ACV-loaded vial + the number of flies in the water-loaded vial) [56]. The PI is hence a value between 1 and −1, with “1” signifying that all responsive flies chose ACV and “−1” signifying that all responsive flies chose water. A PI calculated for a single unit and treatment–sex combination was considered as one observation for further analysis, resulting in 12 replicates for all treatment-sex combinations.

#### 2.3.2. Orientation of Marked Flies by Trap Recapture in the Field

An MRR experiment was executed, to investigate the effect of the mass marking of *D. suzukii* flies with the “Shaking” technique and the Radglo^®^ TP-series fluorescent pigments Orange TP-43 and Blue TP-49 on the overall orientation of the flies in the open field. Recapturing the released flies in this experiment was done by coloured, volatile-attractant-containing traps at different distances from the release point. Hence, if effects on recapture rates were observed, this could be explained by a combination of different factors, including the olfaction, vision, and flight capacity of the flies. The *D. suzukii* culture used in this experiment originated from multiple collections of adults in a private garden (Gentbrugge, Belgium, 51°1.522′ N, 3°46.093′ E) during March and April 2018 and was maintained on a cornmeal-sugar-yeast diet [56] in an incubator (25 ± 1 °C, 60 ± 10% RH, 16:8 h L:D). The experiment ran from 19 October to 9 November 2018 (T_mean_: 7.4 ± 4.4 °C, T_max_: 17.8 °C, T_min_: −3.4 °C, total rainfall: 31.4 mm, Mety (Bodata) weather station at 7.4 km) in a 10 ha plot of the non-host crop *Sinapis alba* (white mustard, not flowering, crop height ca. 30 cm, unmanaged, Landen, Belgium, 50°46.210’ N, 5°03.127’ E).. By selecting this habitat and seasonal timeframe, it was possible to also release unmarked *D. suzukii*: there are very few wild *D. suzukii* in this habitat and any wild drifters would be winter morphs [57] and thus easily distinguished from the laboratory reared summer morphs. In the plot, four trapping grids were deployed with a minimum of 50 m between grids and a minimum of 40 m between grids and plot edges. Each grid consisted of 9 traps (Figure 2B). One trap was placed 1 m north of a central release point, 4 traps at 10 m (one in each cardinal direction), and 4 traps at 20 m (one in each cardinal direction) from the central release point. The trapping devices were red spherical traps (Decis™ Trap Suzukii, experimental prototype, Bayer Crop Science) containing a killing agent [56,58] and an experimental synthetic lure. These traps facilitated assessments as collected flies are dead and dry. Traps were hung on small posts, just above the crop. During three subsequent weeks (7 day interval), 200 marked and 200 unmarked flies were released on each release point. One day prior to release, *D. suzukii* flies of 3–7 days old were selected from the culture and were placed per 100 (sex ratio 50:50) in a vial (Drosophila Container, 68 mL, 36 x 83 mm, PS, Greiner Bio-One) containing a 2 cm layer of cotton wool saturated with 10% sugar solution on the bottom and a spatula tip of dry brewer’s yeast extract (Vitaminor, Natural Granen Gebr. De Scheemaecker NV) and stoppered with a foam stopper (Ceaprenstop, diam. 36 mm, Greiner Bio-One^™^). The flies were handled without anaesthesia, using an aspirator. Next, half of the 16 vials (1600 flies) were marked per vial (100 flies) using the “Shaking” technique: the flies were cold-anesthetised and swirled in a 250 mL glass jar that contained about 20 mg of pigment. After marking, the flies were placed back in their sugar solution/yeast vials for about 24 h in an incubator (25 ± 1 °C, 60 ± 10% RH, 16:8 h L:D) to allow them to groom off excess pigment powder. Unmarked flies underwent a mock treatment without pigment powder. Releasing was done at 1200 h by dumping the flies in a plastic deli container on the soil or on the leaves of the white mustard plants. Collection of the recaptured flies was done at 7 day intervals, at 1100 h. The flies were stored in 70% ethanol (denatured with Eurodenaturant, Disolol^®^, Chem-Lab NV). In the laboratory, within two weeks after sample collection, a stereomicroscope was used to assess morph [56], sex, and mark. UV light (UV compact fluorescent lamp, 25 W, BeamZ or UV 51-LED flashlight, DirectSupply) was used to facilitate the identification of marked specimens. Per grid, the trap counts of each sex-treatment-distance combination were pooled over the duration of the experiment for further analysis.

### 2.4. Persistence of Marks during Trapping or Storage

The persistence of the marking of *D. suzukii* with the Radglo^®^ TP-series fluorescent pigments by the “Shaking” technique under different trapping and storage conditions was assessed; in addition, it was tested how these conditions can affect contaminations between marked and unmarked specimens. Traps for *D. suzukii* typically contain ACV, red wine, or a combination of both as a bait/drowning solution [9,59,60,61,62,63,64,65,66,67,68,69,70,71,72], whereas traps containing lure dispensers can either be dry (if a killing agent is present in the trap, see Section 2.3.2) [56,58,73] or contain water as a drowning solution [64,74,75,76]. Trapped insects are typically stored in ethanol. Hence, five conditions were simulated: dry, water, ethanol (abs. 100% a.r., Chem-Lab NV), ACV (cider vinegar 5% acidity, Burg, Vinaigrerie Fuchs, La Tremblade, France), and red wine (Blygedacht: Merlot-Shiraz-Pinotage, 13.5% Alc. Vol.). Seven-day-old flies (same culture as Section 2.1.) were selected from the rearing and half of them were marked with Radglo^®^ TP-40, using the aforementioned “Shaking” technique and were allowed to groom for seven days in tubes containing a sugar water (10%) saturated plug, the other half remained unmarked but were subjected to the same handling. After the first 24 h of the seven day grooming period, when most excess pigment dust was groomed off, the flies were transferred to new (clean) sugar-water substrate tubes. For each treatment (“dry”, “water”, “ethanol”, “ACV” and “red wine”), five 15 mL centrifuge tubes (transparent PP, with PE screw cap, nerbe plus GmbH) were prepared. A total of 5 mL of the according liquid was added per tube. For the “dry” condition, a 1 cm Ø hole was made in the PE screw caps and covered with stainless steel mesh (100 µm pore) to allow air exchange and prevent condensation and infestation by storage mites. In each tube, five marked and five unmarked flies were placed (cold-anesthetised). All flies died within 24 h. The marked and unmarked flies in the same tube were always of the opposite sex, with three tubes containing marked females and two tubes containing marked males. All individuals were kept in an incubator (25 ± 1 °C, 65 ± 5% RH, 12:12 h L:D). Three times, at a seven-day interval, each tube was shaken and emptied in a Petri dish, after which the flies were sorted in a marked and unmarked group under a stereomicroscope with UV lighting. The identification of marked and unmarked flies was then verified by turning on the visual light source, revealing the sex of the flies and thus enabling unambiguous verification (this procedure is below referred to as the “sorting test”). All flies received a score as described in Section 2.1.2 in order to calculate the marking efficiency index for every marking treatment (marked, unmarked) in each tube and at each assessment time. When the content of a tube had been evaluated, the flies and the liquid were poured back into the tube. After the three assessments (i.e., 7, 14, and 21 days of exposure), the tubes were placed in a dark cabinet at room temperature until eventually a fourth assessment was carried out at 614 days of exposure. In order to further assess the reliability of storing marked flies in ethanol, 12 samples of the field experiment in Section 2.3.2. (i.e., the specimens trapped at 1 m from the release point of each grid at each time point) were re-evaluated after 375 to 389 days: the number of marked flies was counted as described in Section 2.3.2. and then compared with the original observations.

### 2.5. Statistical Analysis

Assumptions of normality were tested using the Shapiro–Wilk test and the histograms were inspected in case of additional assumptions on the distribution. Where relevant, homoscedasticity was verified using the Levene test. For survival analysis (Section 2.1.1 and Section 2.2.), the Kaplan–Meier method with log rank (Mantel-Cox) test was used. In order to compare marking efficiency among treatments (Section 2.1.2), per observation time, a Mood’s median test was performed with pairwise comparisons using the Dunn-test (with Bonferroni correction). To evaluate the persistence of the marking efficiency (Section 2.1.2 and Section 2.4.), per treatment, a Friedman test was executed. If there was sufficient evidence to reject the null hypothesis (i.e., all medians over time are equal), paired Wilcoxon signed-rank tests (with Bonferroni correction) were used post-hoc. The analysis of the trap assays (Section 2.3.1) was done with a two-way ANOVA (type III sum of squares) with “PI” as a variable factor and “sex” and “treatment” (marked/unmarked) as fixed factors. The analysis of the trap recapture in the field (Section 2.3.2) was done per sex and distance from the release point by paired (marked and unmarked flies released in the same grid) two-tailed *t*-tests. To analyse the difference between marked and unmarked flies under the same trapping/storage conditions (Section 2.4.), a paired (marked and unmarked flies stored in the same tube) Wilcoxon signed-rank test was performed per assessment date. To compare counts of marked flies per sample after more than one year of storage in ethanol with those at the initial assessment (Section 2.4.), a paired sign test was conducted. For all analyses the level of significance was set at 0.05. Unless stated otherwise, data provided in the text are always the sample mean ± standard deviation (SD). All statistical analyses were done in IBM SPSS 25.

## 3. Results

### 3.1. Pigment Application Methods

#### 3.1.1. Effect on Survival

**Figure 3:**
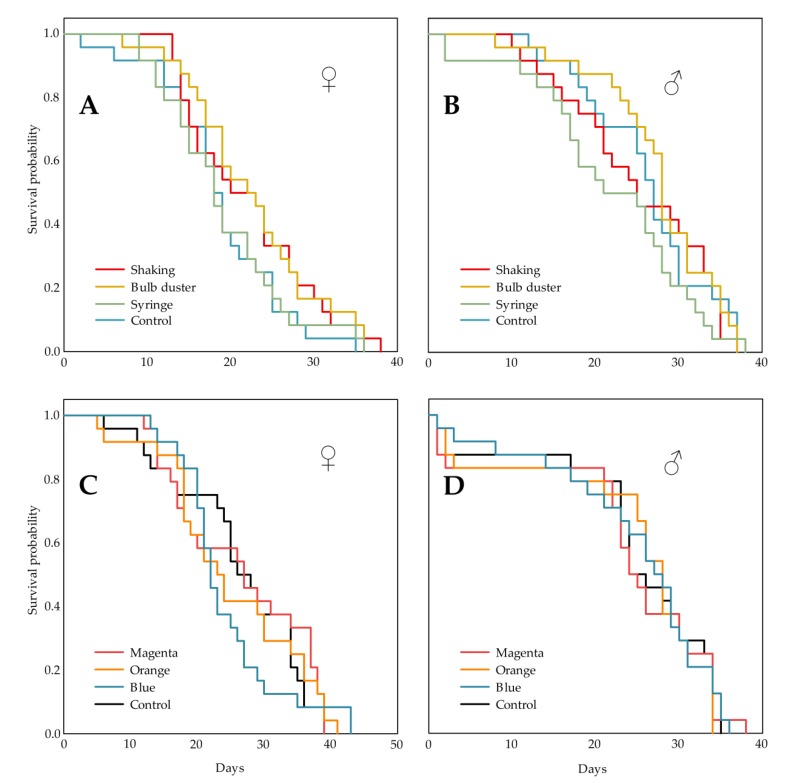
The effect of marking with fluorescent pigments on the survival of *D. suzukii* was investigated. Different application techniques (“Shaking”, “Bulb duster” and “Syringe”) of the pigment (Radglo^®^ TP-40: Chartreuse) and a control treatment were compared, the Kaplan-Meier survival plots are given for females (♀) (**A**) and males (♂) (**B**). For both sexes no statistically significant differences could be denoted. Additionally dyes of the same product series, Magenta (TP-48), Orange (TP-43) and Blue (TP-49), applied by the “Shaking” technique and a control treatment were compared, the Kaplan-Meier survival plots are given for females (♀) (**C**) and males (♂) (**D**). For both sexes no statistically significant differences could be denoted.

Whereas the longest surviving individual of either sex lived up to 46 days, the median longevity was 27 and 35 days for females and males, respectively. The Kaplan–Meier survival plots in Figure 3A,B show that the survival probability of both male and female *D. suzukii* was not altered significantly (χ^2^ (3) = 3.435, *p* = 0.329 and χ^2^ (3) = 4.675, *p* = 0.197, respectively, log-rank test) by marking them seven days after emergence with a fluorescent pigment (Radglo^®^ TP-40: Chartreuse) and that there was no statistical difference between the three marking techniques (“Shaking”, “Bulb duster”, and “Syringe”). There was no censoring of data: no flies escaped during the experiment and the experiment lasted until the last fly died.

#### 3.1.2. Marking Efficiency and Persistence

**Figure 4:**
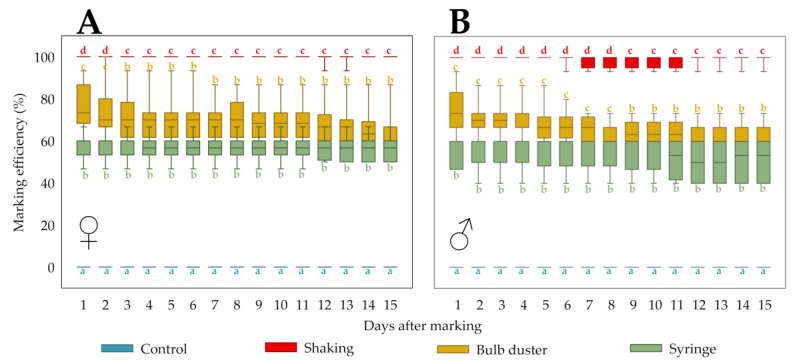
In order to evaluate marking efficiency and persistence of different application methods, the marks of individually kept *D. suzukii* flies were assessed daily. The marking efficiency (%) of each treatment (application techniques and control) is depicted by a box plot for each of the 15 observation days for females (♀) (**A**) and males (♂) (**B**). Different letters denote significant differences between treatments per observation day.

The marking efficiency (%) of each treatment is depicted by a box plot for each observation day in Figure 4A,B for females and males, respectively. Whereas the control had a marking efficiency of 0%, the “Shaking” technique always had a median of 100%. For females, the treatments “Bulb duster” and “Syringe” had median marking efficiencies from 60.0% to 73.3% and from 56.7% to 60.0%, respectively. For males, the treatments “Bulb duster” and “Syringe” had median marking efficiencies from 60.0% to 73.3% and from 50.0% to 60.0%, respectively. Per observation day, the median values were compared (Mood’s median test, pairwise comparisons: Dunn-test, with Bonferroni correction). For the females, on the first two days, all treatments were significantly different (*p* < 0.05 for all contrasts), with “Shaking” > “Bulb duster” > “Syringe” > “Control”. For the males, the same order of differences was observed during the first eight days. During the following 13 (females) and 7 (males) days, the treatments “Bulb duster” and “Syringe” did not differ significantly in terms of marking efficiency, while both were significantly different from the other treatments.

A Friedman test per treatment, evaluating the change in marking efficiency over time (i.e., persistence), showed that at least two time points were significantly different for the treatment “Bulb duster” in females (*p* < 0.0005) and for the treatments “Bulb duster” (*p* < 0.0005) and “Syringe” (*p* = 0.001) in males. However, post-hoc paired Wilcoxon signed-rank tests (with Bonferroni correction) failed to reveal significant differences (*p* > 0.05). Beyond the quantification period of 15 days, regular observations showed that the marking efficiency of all treatments remained similarly constant until the end of the experiment (Section 3.1.1).

### 3.2. Effects of Colour (Dye) on Survival

The longest surviving female and male lived up to 51 and 46 days, with a median longevity of 33 and 34 days, respectively. The Kaplan–Meier survival plots in Figure 3C,D show that the survival probability of both female and male *D. suzukii* was not altered significantly (χ^2^ (3) = 0.712, *p* = 0.870 and χ^2^ (3) = 0.104, *p* = 0.991, respectively, log-rank test) by marking them seven days after emergence with a fluorescent pigment (Radglo^®^ TP-series) and that there is no statistical difference between the three dyes, Magenta (TP-48), Orange (TP-43) and Blue (TP-49). Again, no flies escaped during the experiment and the experiment lasted until the last fly died.

### 3.3. Effects on Behaviour

#### 3.3.1. Olfaction of Marked Flies in Laboratory Trap Assays

**Figure 5:**
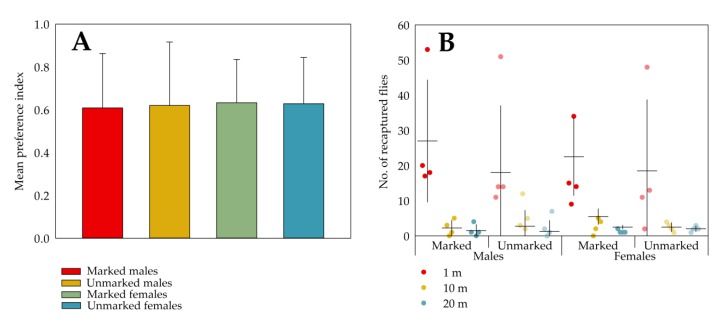
The effects of marking with fluorescent pigments (Radglo^®^ TP-series) using the “Shaking” technique on the behaviour of *D. suzukii* was evaluated both in the laboratory and field. In the laboratory evaluation, for each trap assay unit (n = 12), the preference index (PI) was calculated and means ± SD for each marking-sex combination are shown (**A**). No statistically significant effects of marking, sex or their interaction on the PI could be denoted. For the field evaluation, the numbers of recaptured flies throughout the experiment for each grid are depicted as dots, the means (n = 4) are shown as horizontal lines and the vertical lines indicate the SDs (**B**). For both sexes no statistically significant differences could be denoted between the mean number of marked and unmarked recaptured flies for each distance from the release point.

The mean (n = 12) percentage of responsive flies (i.e., the percentage of the released flies ending up in either of the trap vials after 24 h) was 97.3% ± 4.9% for marked females, 95.8% ± 7.9% for unmarked females, 89.9% ± 10.5% for marked males and 95.9% ± 6.6% for unmarked males. The mean percentage of those responsive flies that chose for ACV was 81.6% ± 10.1% for marked females, 81.4% ± 10.8% for unmarked females, 80.4% ± 12.7% for marked males and 81.0% ± 14.8% for unmarked males. In Figure 5A, the mean preference index (PI) is given for each combination of treatment and sex. The mean PI was around 0.6 in all cases, indicating that all flies were similarly and strongly attracted to ACV. A two-way ANOVA showed that there was no significant effect of treatment (F (1, 44) = 0.002, *p* = 0.961), sex (F (1, 44) = 0.051, *p* = 0.822) or their interaction (F (1, 44) = 0.012, *p* = 0.912) on the PI.

#### 3.3.2. Orientation of Marked Flies by Trap Recapture in the Field

Figure 5B shows the number of recaptured flies (pooled from the three consecutive assessment times) per distance from the release point and per grid. The mean numbers of recaptured flies at 1 m were four- to fifteen-fold higher than at longer distances. As equal numbers of marked and unmarked flies were released in the same grid, the data are paired per grid. This dependency of observations within a grid is notable in the figure, where one of the four grids had a substantially higher number of recaptures of both sexes at 1 m distance than the others. Paired *t*-tests failed to reveal differences between the mean numbers of recaptured marked and unmarked flies, regardless of sex and distance from the release point (males 1 m: t (3) = 2.895, *p* = 0.063; males 10 m: t (3) = 1.111, *p* = 0.348); males 20 m: t (3) = 0.490, *p* = 0.658; females 1 m: t (3) = 0.107, *p* = 0.921; females 10 m: t (3) = 0.147, *p* = 0.893; females 20 m: t (3) = 1.192, *p* = 0.319).

### 3.4. Persistence of Marks during Trapping or Storage

**Figure 6:**
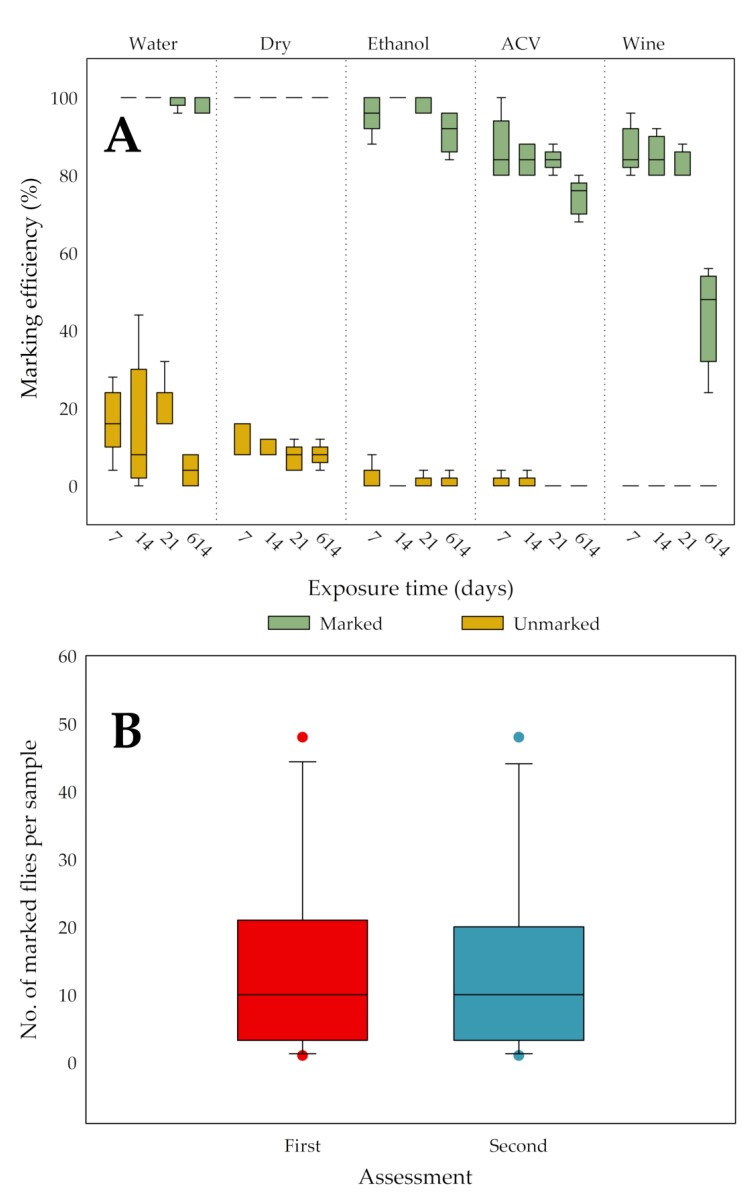
The effect of five different trapping/storage conditions on the marking efficiency (%) of *D. suzukii* flies marked with Radglo^®^ TP-40 (Chartreuse) using the “Shaking” technique was evaluated by storing marked and unmarked flies together in tubes. After 7, 14, 21 and 614 days, all flies in the tubes were scored and for each of these exposure times, box plots per storage condition for each treatment (marked/unmarked) are given (**A**). Statistical analysis showed that per storage condition and exposure time marked and unmarked flies always differed significantly in terms of median marking efficiency. A second assessment of samples containing both marked and unmarked flies, after more than a year of storage in ethanol, resulted in equal median (n = 12) counts of marked *D. suzukii* flies as at the first assessment, after about a week of storage (**B**).

The marking efficiency of marked and unmarked (and possibly contaminated) flies stored under the same conditions as well as the persistence of the marks over time are shown in Figure 6A. For each storage condition and exposure time, marked and unmarked flies differed significantly in terms of median marking efficiency. The results from the “sorting test” followed the same trend: there were no misidentifications or equivocal assignments with the exception of flies stored in red wine for 614 days. In the latter case, flies were darkly stained (almost black) by the wine and hence in three of the five replicates, the “sorting test” failed since not all five marked flies could be correctly assigned. Flies stored in water may be more susceptible to contamination than those stored otherwise and tend to disintegrate, likely due to the hypotonicity of the water. Both ACV and wine stain the flies (brown and red to black, respectively), making it harder (i.e., more UV-light and zoom needed) to see the mark. A Friedman test per storage condition and marking combination, evaluating the change of marking efficiency over time (persistence), showed that at least two time points were significantly different for unmarked flies in water, dry unmarked flies, marked flies in ethanol, marked flies in ACV and marked flies in red wine. However, the post-hoc paired Wilcoxon signed-rank tests (with Bonferroni correction) failed to further prove significant differences between pairs of exposure times. Counts of marked flies in samples (n = 12) with both marked and unmarked flies, which were stored for over a year, were similar to those of the flies in the first assessment, with the exact same median (*p* = 0.125, paired sign test; Figure 6B).

## 4. Discussion

*Drosophila suzukii* is a worldwide economically important pest that currently is only effectively controlled by repeated applications of broad-spectrum insecticides [11,77,78], so the need for more sustainable, integrated solutions is high. In the search for Integrated Pest Management (IPM) tools, an efficient marking methodology can be an important instrument. MRR, MR, and MC studies can be used for assessing (seasonal) dispersal [31,70,73,79,80,81,82] or estimating population sizes [83,84] and can assist in the development of preventative strategies, better targeted control measures or prediction models [85]. MR and MRR studies can also be useful for determining trapping parameters in the development of monitoring [30,86,87,88] and mass trapping strategies [89]. Marking methods can further be applied to (easily) distinguish treatment groups or strains in laboratory, semi-field, or field trials. Another common application of insect marking can be found in SIT programmes [90], a technique that is also under development for *D. suzukii* [91,92,93,94].

Hagler and Jackson [4] define an ideal marking material as “durable, inexpensive, nontoxic (to the insect and the environment), easily applied, and clearly identifiable”.

Fluorescent pigments, like the ones used in this study (Radglo^®^ TP, Radiant Color NV), appear to meet these criteria. These powders made of ground dyed thermoset sulphonamide–melamine–paraformaldehyde resin are durable and are claimed to be lightfast [27,28,95] and resistant to most solvents (like water and alcohols) as well as to high temperatures [26,28,95,96]. These pigments are relatively inexpensive and result in clearly identifiable marks, only requiring (and in some cases not even) an inexpensive UV lamp and/or a dissecting microscope for identification. Thus, fluorescent pigments offer a major advantage over the use of immunomarking, trace elements and stable isotopes in MR and MRR studies. Pigment powders can be easily applied simultaneously on groups of insects by either dusting or some kind of self-marking. The products used in this study are highly similar to other and/or formerly commercialised pigments including the R- and JST-series of Radiant Color NV (Houthalen-Helchteren, Belgium) [97], the A-, AX-, T-, GT- and D-series of Day-Glo Color Corp. (Cleveland Ohio U.S.A.) [27] and the SWADA A-, T-, FEX- and FTX-series of Dane Color UK Ltd. (Stalybridge, UK) [98]. Animal toxicity tests of these melamine-formaldehyde type pigments have shown the products to be non-toxic [28], other effects on the environment are unstudied. These products all have in common that they contain low levels (< 0.1% *w*/*w* [99]) of free formaldehyde, a chemical with toxic properties. While visual marking in general can impede the animals’ camouflage and can both increase or decrease their liability to predation, dusting fluorescent powders is considered inconspicuous and hence most ideal [100].

It is for MR and MRR studies of key importance that the marking technique is evaluated for undesired effects on the longevity and behaviour of the insect [4,99]. Multiple evidence in the literature suggests that such an evaluation should be made for each combination of species, age, fluorescent pigment (series and dye) and application technique/dose. Inorganic “Helecon” pigments (United States Radium Corporation) were found to be indifferent to the survival of *Drosophila pseudoobscura* adults (larval toxicity) [32] but did affect the survival and behaviour of *Cydia pomonella* [101]. Whereas no effect of DayGlo^®^ A-series pigments could be noted on the behaviour of *Synanthedon pictipes* [102], they did affect the survival and behaviour of *C. pomonella* [101]. Dusting *Diabrotica speciosa* with DayGlo^®^ A-series pigments negatively affected its survival, but dusting *Rhyzopertha dominica* with products of the same series did not influence the beetle’s survival or behaviour [103]. Dusting 2–3-day-old *Aedes aegypti* with DayGlo^®^ A-series pigments had an adverse effect on the survival of this mosquito species [20]; conversely, the dusting of 1 or 3-day-old *Anopheles gambiae* adults with a SWADA FTX-series pigment did not impair their survival [19]. The technique of application or the delivered dose of a certain pigment can also result in different effects on insect survival and behaviour. Dominiak et al. [24] reported an effect of the dose (g/L pupae) of a SWADA FEX-series pigment on pupal emergence and behaviour of *Bactrocera tryoni,* whereas Makumbe et al. [25] noted an interaction effect between dose (g/L pupae) and pigment colour on the adult survival of *Bactrocera dorsalis*. Dickens and Brant [20] found that the technique used to dust *A. aegypti* had an impact on both survival and marking coverage. Verhulst et al. [19] showed that dusting *A. gambiae* with a SWADA FTX-series pigment did not affect survival when applied on 1 or 3 day old mosquitoes but did have an effect when applied on 5 or 9 day old mosquitoes. Rojas-Araya et al. [21] reported that the behaviour of *A. aegypti* was altered by dusting with DayGlo^®^ A-series pigments, but not with a DayGlo^®^ ECO-series pigment. The latter is manufactured using a formaldehyde-free resin and has a lower specific gravity than the A-series pigments.

All of the above stress the need for the optimisation of a marking methodology for *D. suzukii* using fluorescent pigments. None of the application techniques used in this study (“Syringe”, “Bulb duster”, and “Shaking”) to mark adult *D. suzukii* with the pigment Radglo^®^ TP-40 adversely affected the survival of female and male flies. The application techniques can be ranked based on the amount (“dose”) of pigment they cover the flies with, the syringe yielding the lowest coverage with its deposition of about 1 mg. The bulb duster resulted in a tenfold deposition, which was comparable to that in the MRR studies on *D. suzukii* of Rice et al. [29] and Kirkpatrick et al. [30], where a deposition of about 6 mg was acquired in a similar way. The “Shaking” technique resulted in the highest level of coverage, with flies being heavily coated. The “Shaking” technique was the most convenient and quick method, followed by the somewhat less convenient “Bulb duster” and the more impractical “Syringe” technique. A 24 h grooming period after marking was always implemented and is needed to allow the flies to remove all hindering excess pigment and to regain their normal behaviour. After this period, even the completely pigment covered flies in the “Shaking” technique were almost not visually marked in visible light (see Figure 1B, score 5). Pigment particles typically cannot be groomed off by the flies between the head and thorax, on the back of the thorax, at the wing bases, and between coxae and thorax. An assessment of the marking efficiency of the different application techniques indicated the clearest mark by the “Shaking” technique. The level of coverage did not change for any application technique during a 15 day observation period after marking and further regular observations strongly indicated that all techniques yielded a lifelong mark. Such a durable mark would imply possible use in MR or MRR studies on long-distance migration or overwintering capacities/behaviour. It is noted that the longevity of the flies in our study is comparable to that in other laboratory studies at similar temperatures and high humidity [104,105,106], even with only sugar water available. In the present study, 3- to 7-day-old flies were marked, which is deemed to be a physiologically relevant age for the use in MRR studies, as the flies are already sexually mature and mated at this point.

Based on the above, it was decided to adopt the “Shaking” technique for all later experiments. No effects on survival were noted for the additional dyes of the same product series: Magenta (TP-48), Orange (TP-43), and Blue (TP-49) and therefore they are assumed to be equally safe for use in marking *D. suzukii*. Multiple colours are often needed in MR, MRR, or other studies, for instance to distinguish released cohorts, treatment groups, or groups originating from different release points. Preliminary experiments indicate that it may be feasible to combine certain (contrasting) colours in order to extend the array of markers, a technique that was earlier described for mosquitoes [107].

Both in laboratory trap assays and in a field recapture study, no differences in behaviour between marked and unmarked male and female flies were recorded. In the trap assays, flies were highly responsive and clearly chose the trap chamber loaded with ACV (a bait known to be preferred by starved *D. suzukii* [56]) regardless of sex and marking. In the field recapture study, similar rates of recapture and dispersal were observed in marked and unmarked flies, which is in line with the results of the field study by Drummond et al. [31].

The persistence of a mark on living flies during a behavioural study is one thing, but it is also desirable that these flies retain their mark in trap reservoirs or in (alcohol) storage (for later assessments) and that there is no transfer (i.e., contamination) of pigment from marked to unmarked flies under these conditions. Our study shows that, in the laboratory, marked flies stored in typical conditions of trapping (dry, or in water, ACV, or red wine) and storage (in ethanol) remained unequivocally identifiable for three weeks. Even an assessment after 614 days yielded similar results as after 7 days, except for red wine, which stains the flies and thus reduces the ability to reliably identify marked flies. As to trapping applications, however, our study presents a worst case scenario considering that in practice flies will never be kept for such long periods in baits or drowning solutions. In contrast, long-term safe storage in ethanol is a relevant and desirable feature. A second assessment of the same samples stored for over a year in ethanol did not result in a significant change in the numbers of correctly identified marked flies. Moreover, under all trapping/storage conditions in the present study, no significant contaminations were noted, not even after 614 days. The “water” condition in our experiment could be considered as a worst case assessment of weather (rain and humidity) effects, and the results indicate that these effects are negligible.

## 5. Conclusions

The present study did not reveal any adverse effects of the marking with fluorescent pigment dusts (Radglo^®^ TP-40: Chartreuse, TP-48: Magenta, TP-43: Orange, and TP-49: Blue) on the survival and behaviour of male and female adults of *D. suzukii,* when marked between day 3 and day 7 of adult life. Effects on behaviour were tested in laboratory trap assays as well as in a field recapture study. The described marking methodology was shown to be convenient, efficient (yielding a highly visible and unambiguously identifiable mark), and highly durable (with a high persistence of the mark on both living flies and flies under trapping/storage conditions), making it highly suitable for mark–(release)–recapture studies. It can be concluded that this study fulfils the need for a thoroughly evaluated methodology for the easy and inexpensive marking of *D. suzukii* that differs from previously described methods [16,18].

## Supplementary Materials

The following are available online at https://www.mdpi.com/2075-4450/11/3/152/s1, Figure S1. A representation of the scores used for the calculation of a marking efficiency index. Scores given ranged from 0 to 5: 0 = no pigment; 1 = traces of pigment; 2 =ungroomed body surfaces homogeneously covered with individual particles—little surface covered; 3 = ungroomed surfaces homogeneously covered with individual particles—more surface covered; 4 = ungroomed surfaces covered with compacted powder (clumps)—little surface covered; 5 = ungroomed surfaces covered with compacted powder (clumps)—more surface covered.
