# Supplementary material for: Marking Drosophila suzukii (Diptera: Drosophilidae) with Fluorescent Dusts
Source: Insects. 2020 Mar 1;11(3):152. doi: 10.3390/insects11030152 (PMC7143264; doi:10.3390/insects11030152)
Supplement: Supplementary file 1 [file insects-11-00152-s001.pdf]

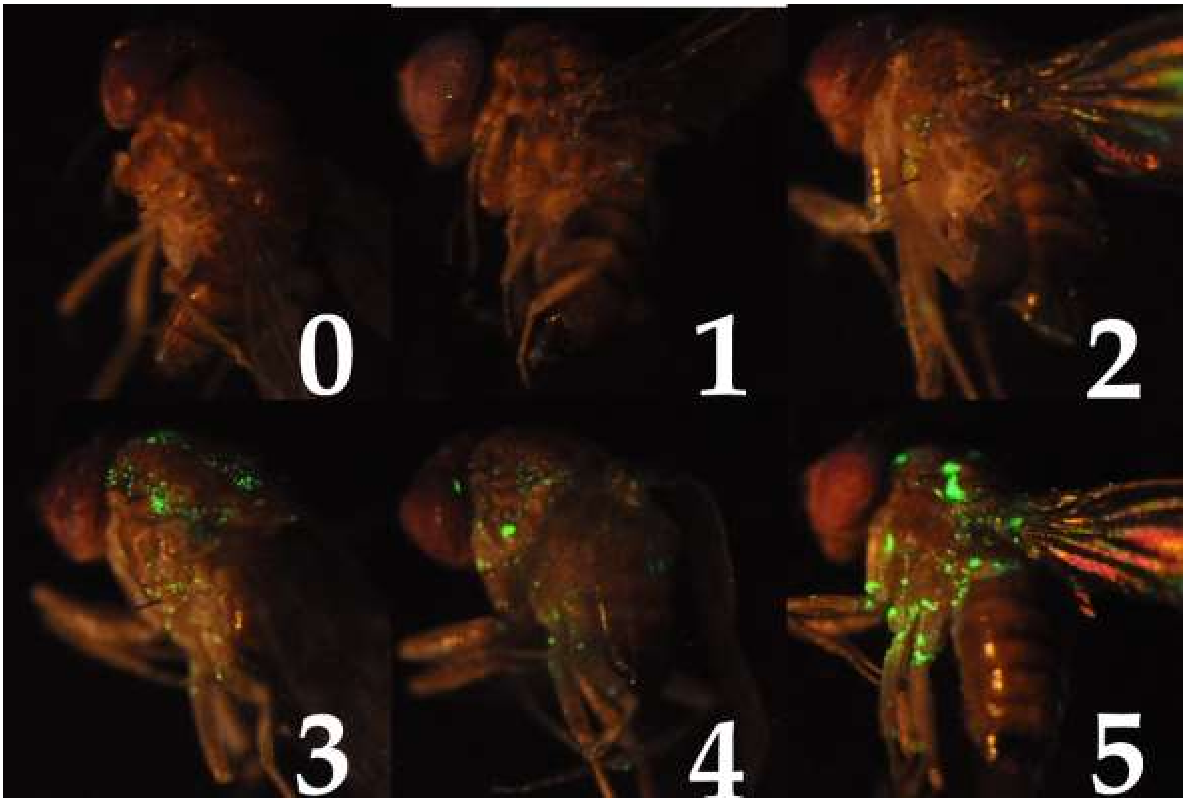

Figure S1. A representation of the scores used for the calculation of a marking efficiency index. Scores given ranged from 0 to 5: 0 = no pigment; 1 = traces of pigment; 2 =ungroomed body surfaces homogeneously covered with individual particles—little surface covered; 3 = ungroomed surfaces homogeneously covered with individual particles—more surface covered; 4 = ungroomed surfaces covered with compacted powder (clumps)—little surface covered; 5 = ungroomed surfaces covered with compacted powder (clumps)—more surface covered.
